# SAMe-TT_2_R_2_ Score in the Outpatient Anticoagulation Clinic to Predict Time in Therapeutic Range and Adverse Events

**DOI:** 10.5935/abc.20170052

**Published:** 2017-04

**Authors:** Fernando Pivatto Júnior, Rafael Selbach Scheffel, Lucas Ries, Ricardo Roitman Wolkind, Roberta Marobin, Sabrina Sigal Barkan, Luís Carlos Amon, Andréia Biolo

**Affiliations:** Hospital de Clínicas de Porto Alegre (HCPA), Porto Alegre, RS - Brazil

**Keywords:** Atrial Fibrillation, Anticoagulants / adverse effects, Decision Support Techniques, Warfarin, Phenprocoumon, Vitamin K

## Abstract

**Background:**

The SAMe-TT_2_R_2_ score was developed to predict which patients on oral anticoagulation with vitamin K antagonists (VKAs) will reach an adequate time in therapeutic range (TTR) (> 65%-70%). Studies have reported a relationship between this score and the occurrence of adverse events.

**Objective:**

To describe the TTR according to the score, in addition to relating the score obtained with the occurrence of adverse events in patients with nonvalvular atrial fibrillation (AF) on oral anticoagulation with VKAs.

**Methods:**

Retrospective cohort study including patients with nonvalvular AF attending an outpatient anticoagulation clinic of a tertiary hospital. Visits to the outpatient clinic and emergency, as well as hospital admissions to the institution, during 2014 were evaluated. The TTR was calculated through the Rosendaal´s method.

**Results:**

We analyzed 263 patients (median TTR, 62.5%). The low-risk group (score 0-1) had a better median TTR as compared with the high-risk group (score ≥ 2): 69.2% vs. 56.3%, p = 0.002. Similarly, the percentage of patients with TTR ≥ 60%, 65% or 70% was higher in the low-risk group (p < 0.001, p = 0.001 and p = 0.003, respectively). The high-risk group had a higher percentage of adverse events (11.2% vs. 7.2%), although not significant (p = 0.369).

**Conclusions:**

The SAMe-TT_2_R_2_ score proved to be effective to predict patients with a better TTR, but was not associated with adverse events.

## Introduction

Vitamin K antagonists (VKAs) reduce the risk for ischemic stroke in patients with atrial fibrillation (AF) by approximately 60%.^1^ The efficacy of the treatment with VKAs is directly related to the time in therapeutic range (TTR), that is, percent time with prothrombin time/international normalized ratio (PT/INR) between 2.0 and 3.0.^2^ A previous study^3^ has suggested that the target TTR would be 58%-65%, below which there appears to be little benefit of oral anticoagulation with VKAs over dual antiplatelet therapy. Additional evidence has emphasized that stroke prevention with the use of VKAs is effective when individual mean TTR is high (> 70%).^4^

Predicting which patients are good candidates for anticoagulation therapy would be very useful. Scores are currently used to assess the risk for thromboembolic events (CHADS_2_ and CHA_2_DS_2_-VASc),^5,6^ as well as the risk for the major adverse effect from that therapy, bleeding (HAS-BLED).^7^ Those scores allow us to assess the indication for that therapy and its risk; however, they provide no information on how the patient will respond to treatment, that is, whether the patient will maintain the target TTR. An easy prediction of which AF patients are likely to reach the target TTR by using VKAs could guide decision making in the strategy of anticoagulation with VKAs or new oral anticoagulants (NOACs).^8^ Recently, Apostolakis et al.^9^ have proposed and validated the SAMe-TT_2_R_2_ score. Those authors have reported the possibility of identifying AF patients on VKAs who reached the target TTR (score 0-1), as well as those who required additional interventions to reach the target TTR, achieving a low TTR with the use of VKAs (score ≥ 2), being thus likely candidates for the use of NOACs. Later studies have validated that score for the prediction of both TTR^8,10-17^ and adverse events.^8,10-12,16,17^ Others, however, have shown that the score cannot do that.^18-20^

In a previous study,^21^ we have described our experience in an outpatient anticoagulation clinic of a Brazilian tertiary hospital, with a mean TTR of 64.8%. This study aimed at describing the TTR according to the SAMe-TT_2_R_2_ score, in addition to relating the score obtained with the occurrence of adverse events in patients with nonvalvular AF on anticoagulation with VKAs.

## Methods

This is a retrospective cohort including patients on oral anticoagulation with VKAs being followed up at the Outpatient Anticoagulation Clinic of the Internal Medicine Service of the Hospital de Clínicas de Porto Alegre (HCPA), a university-affiliated hospital for tertiary care in the Southern region of Brazil. Decisions regarding anticoagulation management were based on the protocol by Kim et al.^22^ All patients attending consultations from January to March 2014 were screened, and those with nonvalvular AF were included in this study. Valvular AF was considered when moderate to severe mitral stenosis or prosthetic heart valve coexisted.^4^

The risk for ischemic stroke was estimated based on the CHADS_2_ and CHA_2_DS_2_-VASc scores, while the risk for bleeding was estimated based on the HAS-BLED score.^5-7^ To analyze the SAMe-TT_2_R_2_ score (0-8 points), the following variables were assessed: female sex (1 point), age < 60 years (1 point), presence of > 2 comorbidities (1 point), use of amiodarone to control heart rhythm (1 point), smoking within 2 years (2 points), and non-Caucasian race (2 points). The following were considered comorbidities: previous stroke, diabetes, peripheral artery disease, coronary artery disease, liver disease, lung disease, kidney disease, hypertension, and heart failure. Patients were categorized based on the SAMe-TT_2_R_2_ score into two groups: low risk (0-1 point) and high risk (≥ 2 points).^9^

Demographic and clinical data and results from complementary tests were obtained via retrospective assessment to electronic medical records, outpatient clinic consultations, visits to the emergency unit and admissions to the HCPA from January to December 2014. Patients lost to follow-up, those who died or whose anticoagulation with VKAs was suspended were also included in the analysis, and the TTR was analyzed up to the last available PT/INR test. Patients were assessed regarding anticoagulation control (via PT/INR tests) and occurrence of adverse events [major bleeding, stroke, transient ischemic attack (TIA), systemic embolism or death]. The TTR was estimated by use of the Rosendaal´s linear interpolation method.^23^

The laboratory tests, left ventricular ejection fraction (preferably assessed on echocardiogram) and number of drugs used were recorded based on the information available on the date closest to the beginning of follow-up. Anemia was considered when hemoglobin (Hb) < 13.0 g/dL for men or < 12 g/dL for women.^24^ Uncontrolled hypertension was defined as systolic blood pressure > 160 mm Hg at the outpatient clinic visit closest to the beginning of follow-up.^7^ Major bleeding was characterized as an event requiring hospitalization or transfusion of red blood cell concentrate, or Hb drop ≥ 2 g/dL.^7^ Kidney disease was considered in the presence of kidney transplantation, chronic dialysis, or serum creatinine ≥ 2.26 mg/dL.^7^ Liver disease was considered in the presence of chronic liver disease (ex.: cirrhosis) or biochemical evidence of significant liver damage (ex.: bilirubin > 2x the upper limit of normality, associated with aspartate aminotranferase, alanine aminotranferase or alkaline phosphatase levels > 3x the normal limit).^7^

### Statistical analysis

Data were analyzed with the *Statistical Package for Social Sciences* (SPSS) software, version 21.0. Descriptive analysis was performed based on the distribution of absolute and relative frequency for qualitative variables, and based on mean ± standard deviation and median for quantitative variables with symmetrical and asymmetrical distribution, respectively. The median 25-75% percentiles were presented when deemed suitable. The groups were compared by using non-paired Student *t* test for symmetrical quantitative variables, Mann-Whitney U test for asymmetrical quantitative variables, and chi-square test for categorical variables. In low-frequency situations, Fisher exact test was used. The normality of the distribution of each variable was assessed by using Shapiro-Wilk test. Area under the *Receiver Operating Characteristic* (ROC) curve was calculated to assess the ability of the SAMe-TT_2_R_2_ score to predict the outcome 'TTR ≥ 65%' and the occurrence of adverse events, the best cutoff point of the score being considered that with the highest sensitivity x specificity product. Event-free survival was assessed by using Kaplan-Meier curves with the Log-Rank test. The significance level adopted for all tests was 5%. This study was submitted to the Committee on Ethics and Research from the HCPA, and approved.

## Results

**Figure 1:**
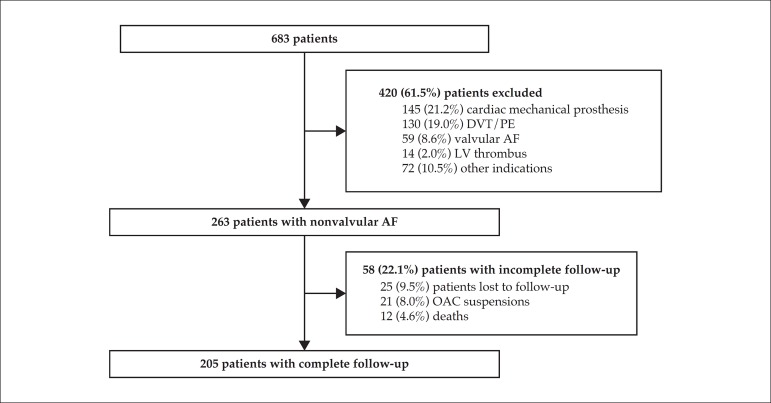
Study diagram. DVT: deep venous thrombosis; PTE: pulmonary embolism; AF: atrial fibrillation; LV: left ventricular; OAC: oral anticoagulation.

This study assessed 263 patients on oral anticoagulation with VKAs due to nonvalvular AF, corresponding to 38.5% of those being followed up at the Outpatient Anticoagulation Clinic of the HCPA. Of those, 205 patients (77.9%) completed the follow-up (Figure 1). Table 1 shows the demographic characteristics of the sample.

**Table 1:** Demographic characteristics of the sample

Variable	n = 263
Female sex	113 (43.0)
Age (years)	71.2 (64.1-78.5)
Use of warfarin	256 (97.3)
Labile PT/INR (TTR < 60%)	124 (47.1)
Hypertension	231 (87.8)
Uncontrolled hypertension	22 (8.4)
HF/LVEF < 40%	149 (56.7)
Diabetes	108 (41.1)
Previous stroke/TIA	96 (36.5)
Coronary artery disease	76 (28.9)
Use of antiplatelet drugs/NSAIDs	64 (24.3)
Anemia	67 (25.5)
Pulmonary disease	36 (13.7)
Previous major bleeding	24 (9.1)
Peripheral artery disease	25 (9.5)
Kidney disease	7 (2.7)
Liver disease	2 (0.8)
Number of medications	7 (6-9)
CHADS_2_	3 (2-4)
CHA_2_DS_2_-VASc	4 (3-5)
HAS-BLED	2 (1-3)

PT/INR: prothrombin time / international normalized ratio; TTR: time in therapeutic range; HF: heart failure; LVEF: left ventricular ejection fraction; TIA: transient ischemic attack; NSAIDs: non-steroidal anti-inflammatory drugs. Categorical variables are shown as n (%), and continuous variables, as median (25%-75%).

During follow-up, 2,754 PT/INR tests (median: 10 tests/patient) were performed, and 1,270 (46.1%) resulted between 2.0 and 3.0. Median TTR was 62.5% (P25-75 44.2%-79.5%). The median of subtherapeutic PT/INR time (< 2.0) was 18.9%, and that of supratherapeutic PT/INR time (> 3.0), 9.6%.

**Figure 2:**
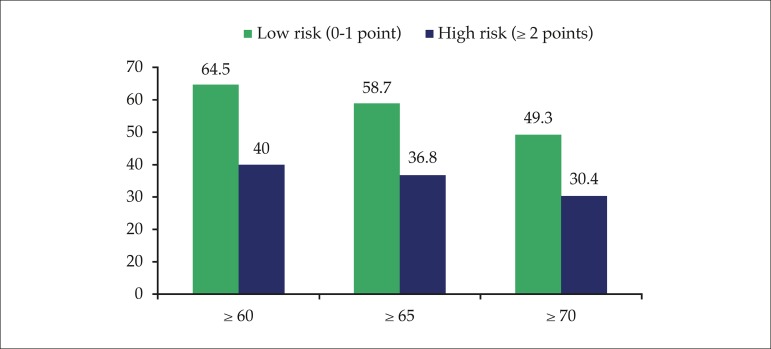
Percentage of patients with TTR ≥ 60%, 65% and 70% according to the points obtained in the SAMe-TT_2_R_2_ score (p < 0.001, 0.001 and 0.003, respectively).

Regarding the SAMe-TT_2_R_2_ score, 138 patients (52.5%) had it 0-1 (low risk), while 125 (47.5%) had it ≥ 2 (high risk), the median being 1 (1-2). When assessing the SAMe-TT_2_R_2_ score criteria individually (Table 2), the criterion "medical history" (presence of > 2 comorbidities) was the most prevalent (57.0%). Low-risk (score 0-1) patients had a significantly higher median TTR as compared to high-risk (score ≥ 2) ones: 69.2% vs. 56.3% (p = 0.002). Likewise, the percentage of patients with TTR ≥ 60%, 65% or 70% was higher among low-risk patients for all cutoff points analyzed (Figure 2).

**Table 2:** Prevalence of the SAMe-TT_2_R_2_ score components

Score Component		n (%)
S	**S**ex (female)	113 (43.0)
A	**A**ge (< 60 years)	41 (15.6)
Me	**Me**dical history (> 2 comorbidities*)	150 (57.0)
T	**T**reatment (amiodarone)	26 (9.9)
T_2_	**T**obacco use (within 2 years)	37 (14.1)
R_2_	**R**ace (non-Caucasian)	22 (8.4)

* Previous stroke, diabetes, peripheral artery disease, coronary artery disease, liver disease, lung disease, kidney disease, hypertension, and heart failure.

**Figure 3:**
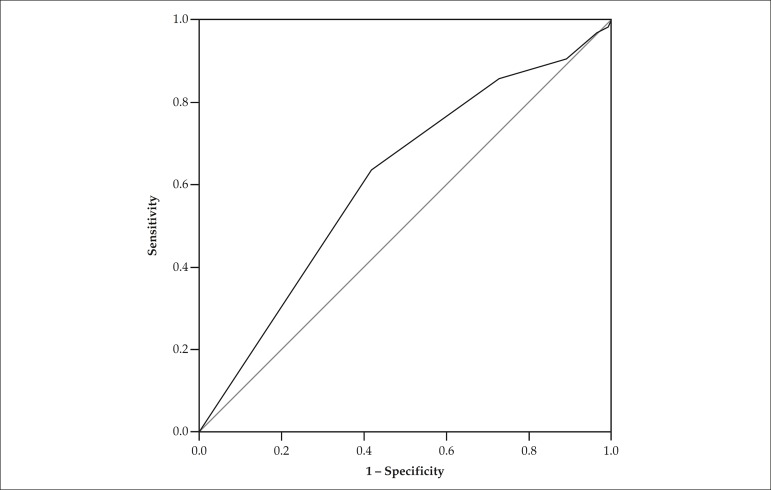
ROC curve for the outcome 'TTR ≥ 65%'.

When assessing the ability of the SAMe-TT_2_R_2_ score to predict the outcome 'TTR ≥ 65%' by using the ROC curve (Figure 3), the cutoff point ≥ 2 showed the best combination of sensitivity and specificity (63.8% and 58.1%, respectively). The area under the curve was 0.612 (95%CI: 0.544 - 0.681; p = 0.002).

**Figure 4:**
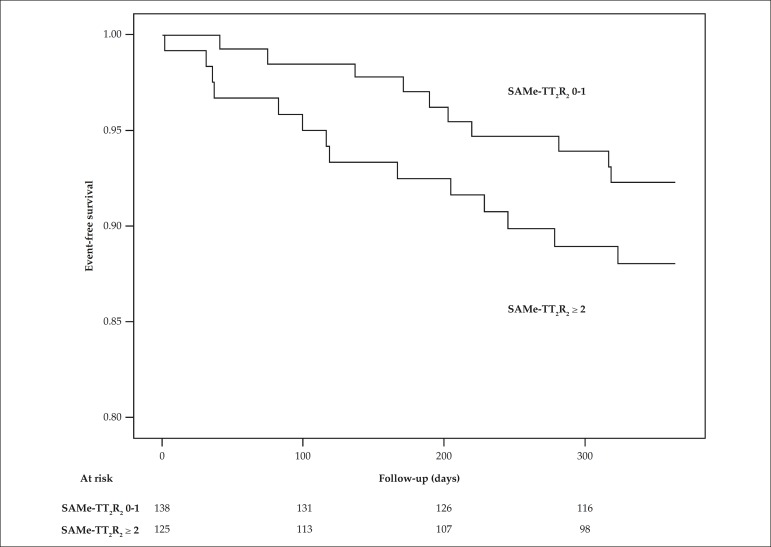
Event-free survival curve according to the points obtained in the SAMe-TT_2_R_2_ score (p = 0.224).

During follow-up, there were 24 (9.1%) adverse events, whose complete description is shown in Table 3. Neither TIA nor systemic embolism occurred during the period studied. High-risk patients (score ≥ 2) had more events, but with no statistically significant difference (11.2% vs. 7.2%; p = 0.369). The area under the ROC curve of the score for the occurrence of adverse events was 0.566 (95%CI: 0.449 - 0.682; p = 0.289), ≥ 2 being again the best cutoff point, with sensitivity and specificity of 58.3% and 53.6%, respectively. Figure 4 shows the event-free survival curves.

**Table 3:** Adverse events in total follow-up and according to the points obtained in the SAMe TT_2_R_2_ score.

Adverse Events	n = 263	SAMe-TT_2_R_2_		p
		0-1 point	≥ 2 points	
Major bleeding	15 (5.7)	6 (4.3)	9 (7.2)	0.465
Stroke	4 (1.5)	1 (0.7)	3 (2.4)	0.349
Death	12 (4.6)	5 (3.6)	7 (5.6)	0.637
TOTAL	24 (9.1)	10 (7.2)	14 (11.2)	0.369

Data shown as n (%).

## Discussion

The use of anticoagulation in patients with AF to prevent thromboembolic events is known to be effective and TTR-dependent. Predicting which patients on VKAs are more likely to reach the target TTR is important, especially currently when new drugs that do not require PT/INR monitoring are available. In this study with a Brazilian sample, the SAMe-TT_2_R_2_ score proved to be a good predictor of TTR for nonvalvular AF patients on oral anticoagulation with VKAs. That score can be useful in the initial assessment of patients with indication for anticoagulation. Median TTR, as well as the percentage of patients with TTR ≥ 60%, 65% and 70%, were higher among patients with a low SAMe-TT_2_R_2_ score (0-1 point) as compared to the group whose score was ≥ 2.

The usefulness of that score in other populations and clinical settings has been reported. Ruiz-Ortiz et al.,^15^ in a prospective analysis of Spanish cardiology outpatients, have reported a progressive decrease in mean TTR according to the score obtained. In their study, patients who scored 0 had a mean TTR of 67.5% ± 24.6%, while those who scored ≥ 4 had a mean TTR of 52.7% ± 28.7% (p < 0.01), with an area under the ROC curve for the outcome 'TTR ≥ 65%' of 0.57 (95%CI: 0.53 - 0.60; p < 0.0005). Roldán et al.,^14^ assessing 459 patients of an outpatient anticoagulation clinic, have reported that those with a score of 0-1 had a mean TTR of 67% ± 18%, while those with a score ≥ 2 had a mean TTR of 61% ± 16% (p < 0.001). In their study, the odds ratio for reaching a TTR < 65% was 2.10 (95%CI: 1.44 - 3.06; p < 0.001) in patients with a score ≥ 2. In a retrospective study including 4,468 patients selected from a registry of primary care units in the United Kingdom, Martinez et al.^17^ have reported that the proportion of patients with TTR ≥ 60% was 44.1% among those with a score of 0-1, and 37.1% among those with a score ≥ 2 (p < 0.01).

The association of the points obtained in the score with the occurrence of anticoagulation adverse events (major bleeding, stroke, systemic embolism and/or death) has been described in other studies^8,10-12,16,17^ after the original study,^9^ always relating the quality of anticoagulation, assessed via TTR, with the occurrence of those outcomes. Only the study by Poli et al.^13^ has not observed that relationship. In a retrospective study including 4,468 AF patients on VKAs with a 3-year follow-up, Martinez et al.^17^ have reported a higher risk for stroke in patients with score ≥ 2 as compared to those with score of 0-1 (log rank p < 0.01). Lip et al.,^12^ in a retrospective study with 8,120 patients (mean follow-up, 1,016 ± 1,108 days), have reported that the SAMe-TT_2_R_2_ score predicted stroke/thromboembolism, severe bleeding and death, reflecting a suboptimum TTR in patients with score ≥ 2. In the present study, the lack of association between the score and the occurrence of adverse events, specifically stroke, can be attributed to the low incidence of that complication.

Several studies have proposed the inclusion of the SAMe-TT_2_R_2_ score in the flowchart for strategic decision-making about which anticoagulant should be used for patients recently diagnosed with AF.^14,25-28^ Based on the score obtained, for patients with ≥ 2 points, the use of NOACs should begin immediately, while those with a score of 0-1 should begin their treatment with VKAs, which should be changed to NOACs if target TTR (> 70%) was not achieved during follow-up. Current guidelines for AF management, however, have not included that strategy.^4,29,30^

Our study has some limitations. Its retrospective design has inherent limitations, which can affect the quality of the data analyzed. Nevertheless, we believe that there was no great loss of data necessary for this study, because at our institution patients undergo systematic care, by use of protocols and structured outpatient clinic visits. Thus, most data necessary for the study was systematically collected during outpatient visits. Another limitation is that the medical record review identified only in-hospital adverse events or events reported by patients during their visits to the outpatient clinic, and some events, especially the adverse ones, might have been missed. Finally, the single-center characteristic of this study ensures the uniform follow-up of the patients described in this cohort, but might have decreased its external validity.

## Conclusion

Based on our findings, the SAMe-TT_2_R_2_ score proved to be effective to predict TTR for AF patients on anticoagulation with VKAs. Thus, the association of that score with the scores to assess the indication of anticoagulation (CHADS_2_ and/or CHA_2_DS_2_-VASc), as well as the risk for bleeding (HAS-BLED), will provide a high-quality assessment of the treatment. For patients with a high SAMe-TT_2_R_2_ score (≥ 2), anticoagulation with VKAs is more likely to be less effective, and, thus, the use of NOACs should be considered. Low-risk patients (score 0-1), however, respond better to VKAs. Therefore, an intervention based on patients' risk allows the use of new technologies (in our case, NOACs), usually more expensive and less available, to be directed to a group of patients with a more specific indication.
